# High expression of *p16^INK4a^* and low expression of *Bmi1* are associated with endothelial cellular senescence in the human cornea

**Published:** 2012-04-03

**Authors:** Ye Wang, Xinjie Zang, Yao Wang, Peng Chen

**Affiliations:** State Key Laboratory Cultivation Base, Shandong Provincial Key Laboratory of Ophthalmology, Shandong Eye Institute, Shandong Academy of medical Sciences, Qingdao, 266071 China

## Abstract

**Purpose:**

Determine cyclin-dependent kinase inhibitor 2A (*p16^Ink4a^*) and polycomb ring finger oncogene (*Bmi1*) expression in corneal endothelium samples from different age groups and test whether the expression of *p16^INK4a^* and *Bmi1* are associated with endothelial cellular senescence in human cornea.

**Methods:**

Samples were selected from an eyebank of healthy human corneal endothelial cells (HCECs). Donor human corneas were divided into three age-groups: age ≤30 years, 30–50 years and ≥50 years. The expression of *p16^INK4a^* and *Bmil* were analyzed by real-time PCR, immunohistochemistry, and immunofluorescence.

**Results:**

Through real-time PCR, we detected less than threefold decreases in *Bmi1* expression and greater than fivefold increases in *p16^INK4a^* expression associated with aging. *Bmi1* expression was significantly down-regulated with increasing donor age. The number of *p16^INK4a^*-positive cells was significantly higher and the number of *Bmi1*-positive cells was significantly lower in older donors compared to the younger age groups. Our immunohistochemistry experiments showed that the expression of *p16^INK4a^* in older donors was stronger than that in younger donors and the expression of *Bmi1* in older donors was weaker than that in younger donors. Results from both the immunohistochemistry and real-time PCR experiments confirmed increased expression of *p16^INK4a^* and decreased expression of *Bmi1* with age in HCECs. Additionally, the results of immunofluorescence double-staining for *p16^INK4a^* and *Bmi1* further validated the immunocytochemistry and real-time PCR results.

**Conclusions:**

Our data are the first to demonstrate that high expression of *p16^INK4a^* and low expression of *Bmi1* are associated with endothelial cellular senescence in human cornea. Our findings are not just for cornea transplantation but also for a better understanding of the cornea senescence and the process of aging in this sepcific human organ.

## Introduction

In clinics, the evaluation of the quality of donor corneas is very important for corneal transplantation. In particular, the evaluation of the donor cornea is heavily dependent on the evaluation of human corneal endothelial cells (HCECs). Although the significance of cyclin-dependent kinase inhibitor 2A (*p16^Ink4a^*) in cell senescence in most tissues is known, its importance in HCECs unknow. The quality of donor corneal endothelial cells is very important for transplant success. Studies in model organisms have suggested that the long-term persistence of donor-derived endothelial cells may be a necessary condition for graft transparency and long-term survival [1-3]. Due to the lack of proliferative capacity of these cells in vivo, the importance of donor corneal endothelial cell health in corneal transplantation is generally agreed upon. The fact that a significant number of corneas from elderly donors maintain excellent function for a prolonged period of time suggests that biologic age rather than chronological age determines corneal quality and long-term functionality after transplantation. Pioneering work has shown that HCECs are arrested in the G_1_ phase of the cell cycle and do not proliferate ex vivo [4]. Due to this relative lack of cell division, human endothelial cell density (ECD) in the normal, healthy cornea decreases with age [5-7]. The reason remains unclear.

Many studies have shown that cell senescence is closely related to the expression of the inhibitor of cyclin-dependent kinase *p16^INK4a^* [8-11]; this inhibitor has received much attention due to its ability to mediate senescence-associated growth arrest [12-14], and inhibition of *p16^INK4a^* plays a decisive role in regulating G_1_ arrest. In most animal and human tissues, *p16^INK4a^* has been shown to markedly increase with aging and can serve as a biomarker of tissue aging [15-17]. Work in our laboratory has shown an age-related increase in *p16^INK4a^* expression in normal HCECs in vivo [18]. One important pathway involved two cyclin-dependent kinase inhibitors, *p16^INK4a^* and cyclin-dependent kinase inhibitor 2A (mouse; *p19^Arf^*) is regulated by polycomb ring finger oncogene (*Bmi1*), the first identified polycomb gene family member, which plays important roles in cell cycle regulation, cell senescence, and cell immortalization. The close correlation between down-regulation of *p16^INK4a^* and the upregulation of *Bmi1* has been proved in lung cancer and neuroblastoma tumors [19]. Currently there has been no report about the expression of *Bmi1* in HCECs. Therefore, in the present study, we aimed to determine *Bmi1* expression in corneal endothelium samples from different age groups and test whether the expression of *p16^INK4a^* and *Bmi1* are associated with endothelial cellular senescence in human cornea. In this report, we have analyzed the expression of *p16^INK4a^* and *Bmi1* in healthy human corneal endothelium biopsies taken from donors in various age groups. We believe this is a first step toward a better assessment of the health of donor corneas and of the suitability of corneal organs for transplantation.

## Methods

The handling of donor tissues was consistent to the tenets of the Declaration of Helsinki of 1975 and its 1983 revision in protecting donor confidentiality.

### Human corneal tissue

**Figure 1:**
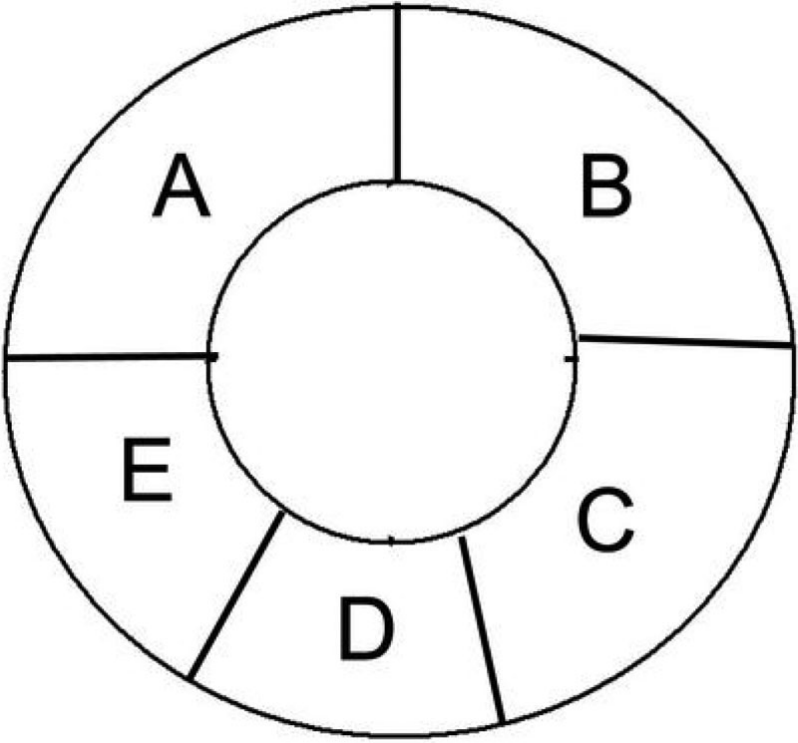
The corneal rims were divided into five parts (A to E). Part A was used for immunofluorescence staining. Part B was stained with trypan blue and alizarin red. The endothelium from part C was stripped away intact and frozen at −80 °C for RNA analysis. Part D and part E were used for the immunohistochemical staining of p16^INK4a^ and Bmi1, respectively.

This study was approved by the Shandong Eye Institute Review Board. The human donor corneas were provided by the International Federation of Eye Banks, Eye Bank of Shandong China (Qingdao, China) and preserved in DX intermediate-term medium at 4 °C [18,20]. Most of the donors had causes that did not comprise corneal or eye disease. The death-to-preservation interval was less than 30 h. Corneas were accepted only if the donor history and condition of the corneas indicated no damage of the endothelium. The donor corneal rims (residual tissues) used for this study were collected via penetrating keratoplasty. The corneal rim tissues were placed with the endothelium side up on a Teflon block under a surgical microscope, and the tissues were cut (with a 10–11 mm-diameter circular trephine) along the Schwalbe’s line. The corneal rims were divided into five parts (part A to part E; Figure 1). Part A was used for immunofluorescence staining. Part B was stained with trypan blue and alizarin red [21]. For part C, Descemet’s membrane along with the endothelium was stripped away, intact, from the underlying stroma using forceps. The stripped endothelial tissues were frozen at −80 °C for RNA analysis. Parts D and E were used for immunohistochemistry of *p16^INK4a^* and *Bmi1*, respectively. Donor information and corneal endothelial cell density are listed in Table 1.

**Table 1:** Donor information and corneal endothelial cell density (cells/mm^2^, OS/OD).

**Groups**	**Donor age**	**Density (cells/mm^2^; OS/OD)**	**Death to preservation time (h)**
	3	3200/3200	11
	5	3210/3340	8
	15	2900/2560	11.5
	20	3120/3080	20
age≤30y	21	3120/3160	5
	21	3220/3180	20
	22	3080/3140	12
	23	3220/3180	8
	24	2900/2920	4
	25	2980/3020	21.5
	26	2920/2880	23
average		3080/3060	
	32	2780/2760	26.5
	35	2700/2720	2.5
	37	2700/2760	23
	37	2780/2760	3
	37	2780/2820	21.5
30y<age<50y	37	2820/2860	21
	40	2860/2800	24
	45	2680/2700	3.5
	45	2600/2680	8
	45	2680/2720	25.5
	49	2760/2740	27.5
average		2740/2756	
	51	2500/2540	24
	51	2620/2580	25.5
	51	2580/2600	24
	52	2480/2540	26
age≥50y	55	2350/2400	26
	55	2700/2680	21
	57	2480/2400	27.5
	61	2420/2400	26
	62	2260/2200	27.5
	68	2420/2440	23
	77	2120/2070	26.5
average		2450/2440	

### *P16* promoter constrcts and luciferase assays

*p16^INK4a^* promoter constrcts and overexpression of *Bmi1* were performed according to previously described reference [22]. Briefly, the 1.2 Kb *p16^INK4a^* promoter was amplified from corneal endothelium of a 67-year-old donor and cloned into the promoterless luciferase plasmid pGV-B (Toyo Ink, Tokyo, Japan). The cytomegalovirus (CMV) promoter was also cloned into the plasmid pGV-B. The deletion mutants of the Bmi1 were cloned into pCDNA3.1 vector with BamHI and XhoI sites. *Bmi-1* shRNA plasmids (sc-29814-SH) and control shRNA plasmids (sc-108060) were obtained from Santa Cruz Biotechnology (Santa Cruz, CA). *p16^INK4a^* promoter luciferase assays were performed with the luciferase assay kit (Promega, Mannheim, Germany) according to the manufacturer’s protocol. Forty-eight hours after transfection, HCECs were collected and lysed using 50 µl of lysis buffer. These expriments were performed in triplicate and repeated at least twice for confirmation.

### Real-time quantitative PCR

Total RNA was isolated according to the manufacturer’s protocol (NucleoSpin RNA II System; Macherey-Nagel, Düren, Germany) and subjected to reverse transcription at 42 °C for 60 min in a 40-µl reaction mixture using a first-strand cDNA synthesis kit (BBI, Toronto, ON, Canada). The reagents (TaqMan; Applied Biosystems, Foster City, CA) and sequence detection system (ABI Prism 7500 System; Applied Biosystems) were employed in real-time PCR as recommended by the manufacturer. Each sample was assayed in duplicate (TaqMan Universal PCR Master Mix; Applied Biosystems). The primers and oligonucleotide probes used are listed in Table 2. Cycling conditions were as follows: 10 min at 95 °C followed by 40 cycles of amplification for 15 s at 95 °C and 1 min at 60 °C. The expected fragment length was between 150 and 300 bp. Quantification data were analyzed with SDS system software (7500 System; Applied Biosystems). The log-linear portion of the fluorescence versus cycle plot was extended to determine a fractional cycle number at which a threshold fluorescence was obtained (threshold cycl [23]), and this number was used as a reference for each analyzed gene and for glyceraldehyde-3-phosphate dehydrogenase (*GAPDH*).

**Table 2:** Primers used for real-time PCR.

**Gene name**	**Primer sequence**	**Probe sequence**	**Product length**
*p16^INK4A^*	F: cttcctggacacgctggt	gacctggctgaggagctg	162 bp
(NM_000077)	R: gcatggttactgcctctggt		
*Bmi1*	F: ccagggcttttcaaaaatga	accagaacagattggatcgg	271 bp
(NM_005180)	R: gcatcacagtcattgctgct		
*Ki67*	F: gatcccatttctggggattt	tgaatctcccttttggaagc	264 bp
(NM_002417)	R: ggtctccccctgtaaaccat		

### Immunohistochemistry and immunofluorescence

Corneal samples were fixed for 10 min in 1 mk of cold (−20 °C) methanol and rinsed three times in phosphate-buffered saline (PBS). The samples were then permeabilized in 1.0% Triton X-100 (Sigma-Aldrich, Shanghai, China) in PBS for 10 min at room temperature, followed by incubation with 5% BSA (Boster Biologic Technology, Ltd., Wuhan, China) in PBS for 10 min at room temperature to block nonspecific binding. For immunohistochemistry, the corneal samples were subjected to staining using the EliVision^™^ plus kit (Maxim Corp, Fuzhou, China) according to the manufacturer’s protocol. A color reaction was detected using a diaminobenzidine (DAB) kit (Boster Biologic Technology, Ltd.). For immunofluorescence, the corneal pieces were incubated in a mixture of the two primary antibodies at an appropriate dilution for 2 h at room temperature. After the samples were washed in PBS, they were placed in a mixture of two corresponding fluorescence-conjugated secondary antibodies for 30 min at room temperature. The samples were then placed, endothelial side up, on slides in mounting medium containing DAPI for nuclear staining (Sigma-Aldrich, Shanghai, China). The primary antibodies used were a mouse monoclonal anti-p16^INK4a^ antibody (1:300; SC-1661), a rabbit polyclonal anti-Ki67 antibody (1:300; SC-15402), and a goat polyclonal anti-Bmi1 antibody (1:300; SC-8906; Santa Cruz Biotechnology). Depending on the primary antibody used, the secondary antibody used was rhodamine (TRITC)-conjugated AffiniPure goat anti-mouse IgG (H+L; 1:200; ZF0313; zsbio, Beijing, China) or donkey anti-goat IgG-FITC (1:100, SC-2024; Santa Cruz Biotechnology). Additionally, isotype control antibodies were used at the same concentration as the primary antibodies. The isotype control antibodies used were mouse IgG (SC-2025) and goat IgG (SC-2028; Santa Cruz Biotechnology). Digital images were obtained using an Eclipse C1si Spectral Imaging Confocal Microscope (Nikon Instruments Inc. Melville, NY).

### Senescence-associated β-galactosidase (SA-β-Gal) activity staining

SA-β-gal activity staining was performed using SA-β-gal staining kit (Beyotime Institute of Biotechnology, Haimen, China) according to the manufacturer’s protocol. Corneal whole mounts were washed with PBS, endothelial cell side up, and fixed with 4% formaldehyde. After washing with PBS (pH 6.0), the tissues were incubated at 37 °C overnight in a humidified chamber with freshly prepared SA-β-Gal staining solution. On the following day, tissues were washed twice in PBS at room temperature for 10 min, and staining was visualized and captured using a microscope equipped with a digital camera (Eclipse e800; Nikon).

### Statistical analysis

The differences in numbers of positively stained cells between all age groups were analyzed using a Kruskal–Wallis non-parametric ANOVA, and differences between single age groups were analyzed using the Mann–Whitney U test. The correlation between donor age and the expression of *p16^INK4A^* was tested using simple linear regression models. R-squared statistics and Pearson correlations were calculated (SPSS software version 12.0; SPSS Inc., Chicago, IL). A p-value of less than 0.05 was considered to be significant.

## Results

### Regulation of *p16^INK4a^* promoter activity by *Bmi1* in HCECs

**Figure 2:**
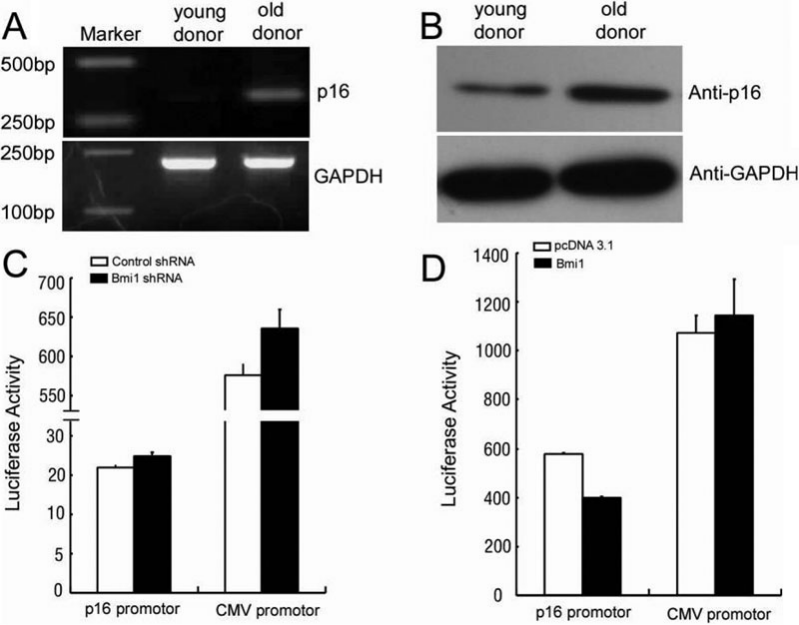
*Bmi1* can regulate the activity of the *p16^INK4a^* promoter. The mRNA expression of *p16^INK4a^* were determined by reverse transcription-PCR (**A**) and the protein expression of p16^INK4a^ were determined by western blot (**B**). *p16^INK4a^* promoter or CMV promoter cloned into Luciferase vector were transfected into HCECs from young donor separately along with pCDNA3.1-*Bmi1* or pCDNA3.1 (**C**). *p16^INK4a^* promoter or CMV promoter cloned into luciferase vector were transfected into HCECs from old donor separately along with *Bmi1*-shRNA or control vector (**D**). Luciferase activity was measured 48 h after transfection.

The expression of *p16^INK4a^* was much higher in HCECs from old donor (67 years old) than that in HCECs from young donor (21 years old; Figure 2A,B). Then we examined whether the activity of *p16^INK4a^* promoter could be regulated by *Bmi1*. For overexpression of *Bmi1*, HCECs from old donor were transfected with pCDNA3.1-*Bmi1*. For knockdown of *Bmi1*, HCECs from young donor were transfected with *Bmi1*-shRNA. The results showed that *p16^INK4a^* promoter activity decreased when *Bmi1* was overexpressed and *p16^INK4a^* promoter activity increased when *Bmi1* was knockdown. However, there was no effect on CMV promoter activity when *Bmi1* was overexpressed or knockdown (Figure 2C,D). These results indicated that *Bmi1* could regulate the activity of the *p16^INK4a^* promoter.

### Expression of *p16^INK4a^* in HCECs Is upregulated with aging

**Figure 3:**
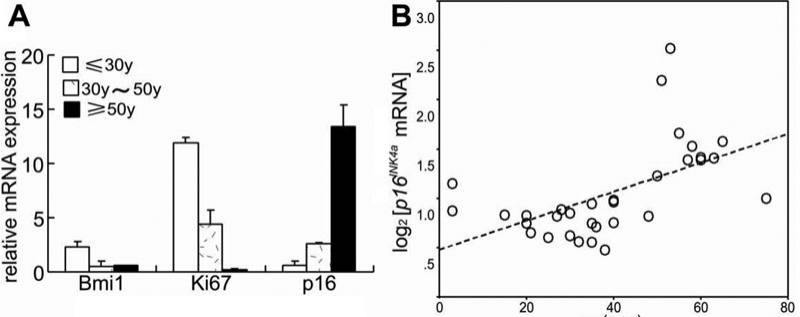
Results of real time PCR. Expression of *Bmi1*, *Ki67*, and *p16^INK4a^* in HCECs from donors of different ages analyzed by real-time PCR (**A**). There was a statistically significant difference in *p16^INK4a^* expression between the donors of younger than 30 years and older than 50 years of age (p≤0.001). There was also a statistically significant difference in *Ki67* expression between donors of younger than 30 years and donors older than 50 years of age (p≤0.001). The figure depicts a Pearson correlation of *p16^INK4a^* gene expression with age (r=0.560, p=0.001; **B**).

To verify that *p16^INK4a^* can serve as a biomarker of endothelial cellular senescence in the human cornea, we first measured the expression of *p16^INK4a^* in HCECs by real-time PCR. Figure 3A shows the expression of *p16^INK4a^* normalized to *GAPDH* and the fold change in gene expression. There was a statistically significant difference in *p16^INK4a^* expression between the donors younger than 30 years and older than 50 years of age (p≤0.001). Antigen identified by monoclonal antibody Ki-67 (Ki67) is a cellular marker that is closely associated with cell proliferation. We also detected the expression of *K_i_-67* in HCECs, and there was a statistically significant difference between the donors younger than 30 years and older than 50 years of age (p≤0.001).

**Figure 4:**
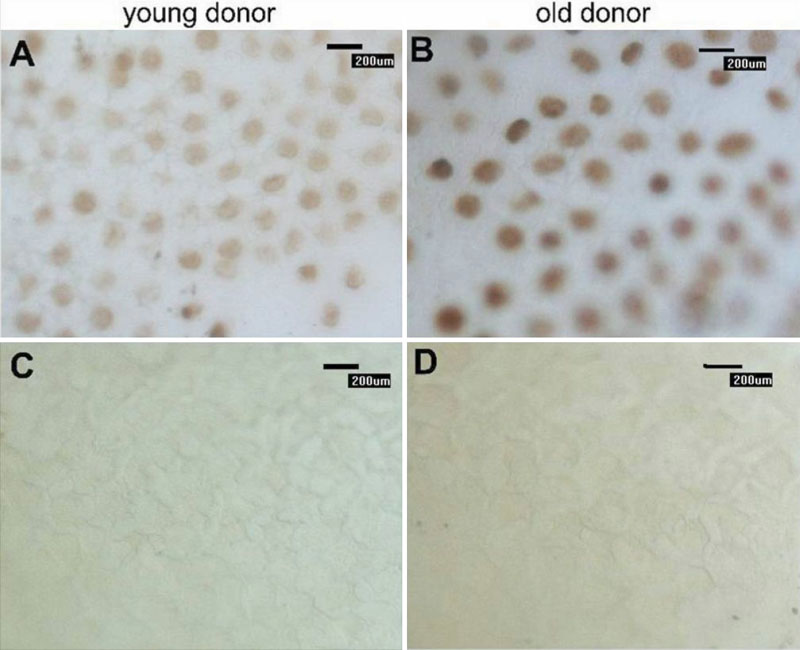
p16^INK4a^ expression was detectable and showed similar nuclear localization in corneal endothelial cells from donors of different ages. The strength of p16^INK4a^ expression in a young donor (**A**) was stronger than that in an elderly donor (**B**). Panels **C** and **D** represent the negative control. Magnification: 200×.

**Figure 5:**
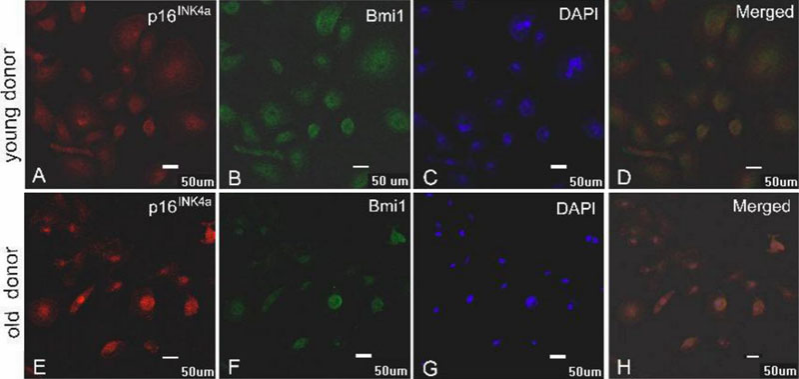
Results of double-staining of p16^INK4A^ and Bmi1. Immunofluorescence double-staining of p16^INK4A^ (red) and Bmi1 (green) in samples from a donor younger than 30 years of age (**A**-**D**) and from a donor older than 50 years of age (**E**-**H**). Panels **C** and **G** represent DAPI staining.

Representative results of our immunohistochemistry experiments are displayed in Figure 4. *p16^INK4a^* expression was detectable in corneal endothelial cells from donors of different ages. The positive staining observed showed a similar pattern of nuclear localization in the HCECs in cornea tissues from donors of different ages. These results revealed a significant upregulation of *p16^INK4a^* expression in HCEC biopsies as the donor age increased. As shown in Figure 3B, we found a strong correlation (Pearson) of *p16^INK4a^* gene expression with age (r=0.560, p=0.001). Additionally, the immunofluorescence results, as presented in Figure 5A,E, revealed an upregulation of *p16^INK4a^* expression in HCECs. These data suggest that, in vivo, *p16^INK4a^* expression is upregulated with increasing age of HCEC donors, indicating that the expression of *p16^INK4a^* maybe serves as a biomarker of senescence in human corneal endothelium similar to its function as a biomarker in other mammalian tissues [17,24-29].

### Age-dependent upregulation of *p16^INK4a^* is associated with down-regulation of *Bmi1*

**Figure 6:**
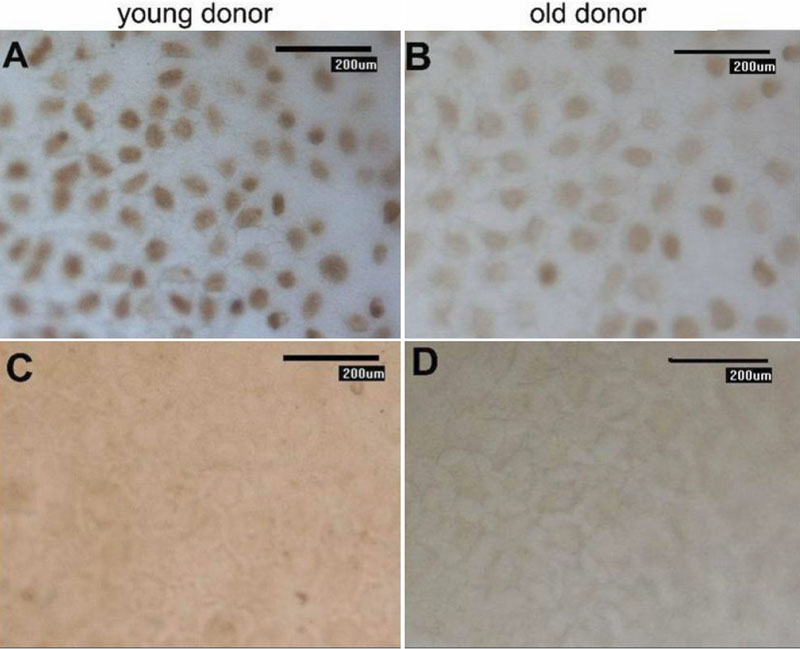
Immunohistochemistry of Bmi1 expression. A large number of Bmi1-positive cells were detectable in corneal endothelial cells from donors younger than 30 years of age (**A**). In contrast, Bmi1 expression was rarely detectable in corneal endothelium biopsies from donors older than 50 years of age (**B**) and the number of Bmi1-positive cells were noticeably decreased compared to samples from younger donors. Additionally, we found no staining in the negative control samples (**C** and **D**), indicating that the staining was specific. Magnification: 200×.

*Bmi1* is a member of the polycomb group of transcriptional repressors that was initially identified as an oncogene cooperating with c-myc in a murine lymphoma model [30]. *Bmi1* is a negative regulator of *p16^INK4a^* gene expression [31]. To address the question of whether *Bmi1* is related to HCECs aging in vivo, we evaluated the expression of *Bmi1* by Real-Time PCR. Figure 3 shows the expression of *Bmi1* normalized to *GAPDH* and the fold change in gene expression. There was a statistically significant difference in *Bmi1* expression between the donors younger than 30 years and older than 50 years of age. (p≤0.001). By immunohistochemistry, we found that a large number of Bmi1-stained cells were detectable in corneal endothelial cells from donors younger than 30 years of age (Figure 6A). In contrast, *Bmi1* expression was rarely detectable in corneal endothelium biopsies from donors older than 50 years of age (Figure 6B), and the number of Bmi1-positive cells was considerably decreased. Additionally, we found no staining in a negative control (Figure 6C,D), indicating that the staining was specific for Bmi1.

**Figure 7:**
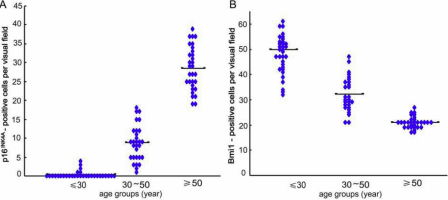
p16^INK4a^ and Bmi1 staining of three biopsies from each age group, shown as the number of positive cells counted in a total 10 visual fields for each sample. The average number of p16^INK4a^ positive cells per field was less than 5 in donors younger than 30 years of age, 10 in donors older than 30 of years and younger than 50 years of age, and 30 in donors older than 50 years of age (**A**). The number of Bmi1 positive cells was determined in the same way. The average number of Bmi1-positive cells per field was 50 in donors younger than 30 years of age, 30 in donors older than 30 of years and younger than 50 years of age, and 20 in donors older than 50 years of age (**B**).

Pioneering work has shown that in human skin, the expression of *Bmi1* is correlated with *p16^INK4a^* [16]; therefore we sought to determine whether the expression of *Bmi1* is also correlated with *p16^INK4a^* in HCECs. We selected three biopsies from each age group for staining for p16^INK4a^ and Bmi1. For each biopsy, the number of positive cells was counted in a total of 10 visual fields. The average number of p16^INK4a^-positive cells per field was less than five in donors younger than 30 years of age, less than 10 in donors between 30 and 50 years of age, and less than 30 in donors older than 50 years of age (Figure 7A).

The number of Bmi1-positive cells was determined in the same way. The average number of Bmi1-positive cells per field was 50 in donors younger than 30 years of age, 30 in donors between 30 and 50 years of age, and 20 in donors older than 50 years of age (Figure 7B). These data indicate a significant upregulation of *Bmi1* expression in the HCECs of donors younger than 30 years of age correlated with a significant down-regulation of *p16^INK4a^* expression. The expression of *p16^INK4a^* was negatively correlated with the expression of *Bmi1*. The promoters of the INK4A locus genes *p16^INK4A^* and *p19^ARF^* are downstream targets of *Bmi1*, which indicates that the age-related down-regulation of *Bmi1* likely contributes to the age-related upregulation of *p16^INK4A^*. To further validate our conclusions, we performed immunofluorescence double-staining for p16^INK4a^ and Bmi1. Figure 5 shows that the fluorescence intensity of p16^INK4a^-positive cells (red) in samples from donors older than 50 year of age was stronger than that of samples from donors younger than 30 years of age (Figure 5A,E). However, the fluorescence intensity of cells positive for Bmi1 expression (green) in samples from donors older than 50 years of age was lower than that in samples from donors younger than 30 years of age (Figure 5B,F). The results of double-staining for p16^INK4a^ and Bmi1 are displayed in Figure 5D,H.

**Figure 8:**
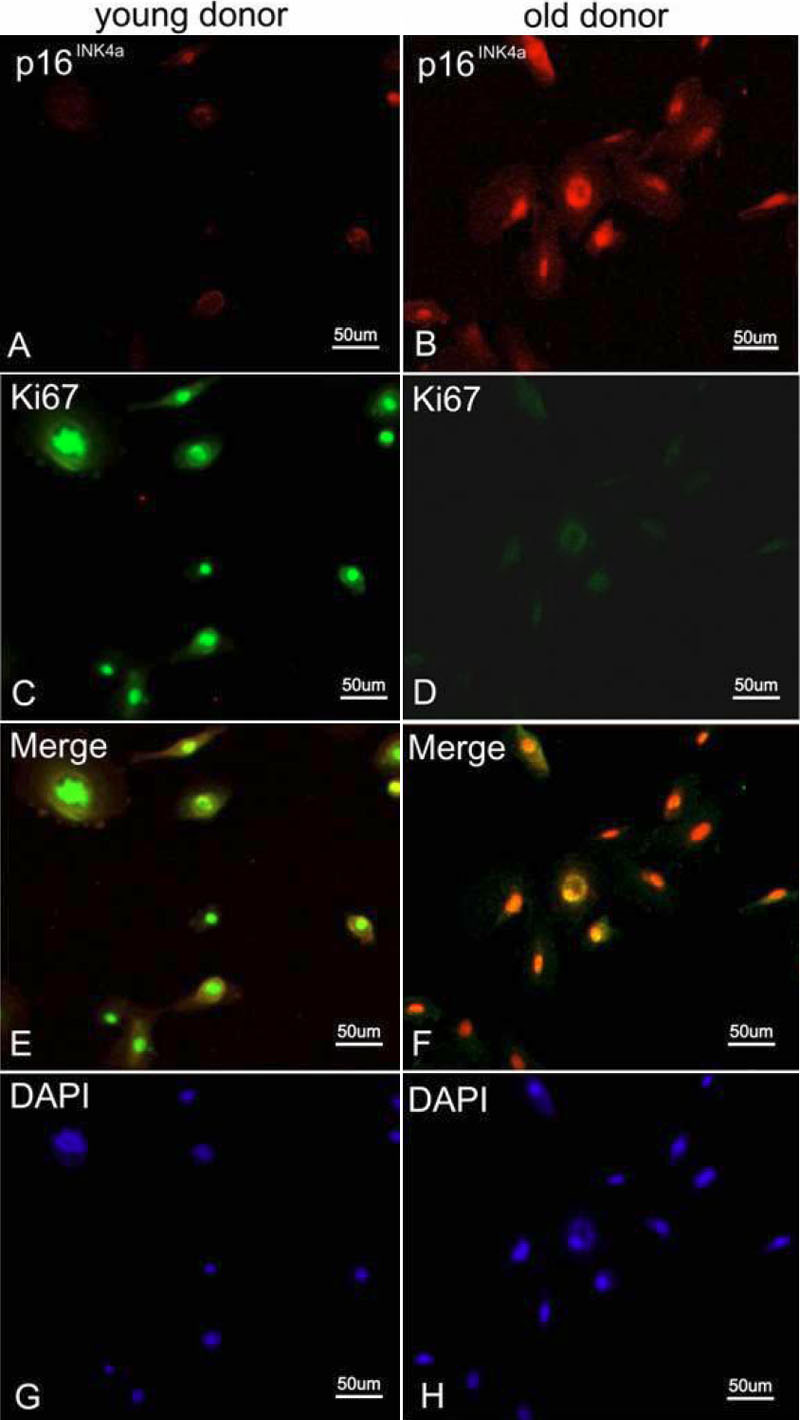
Results of double-staining of p16^INK4A^ and Ki67. Immunofluorescence double-staining of p16^INK4A^ (red) and Ki67 (green) in samples from a donor younger than 30 years of age (**A**, **C**, and **E**) and from a donor older than 50 years of age (**B**, **D**, and **F**). Panels **G** and **H** represent DAPI staining.

**Figure 9:**
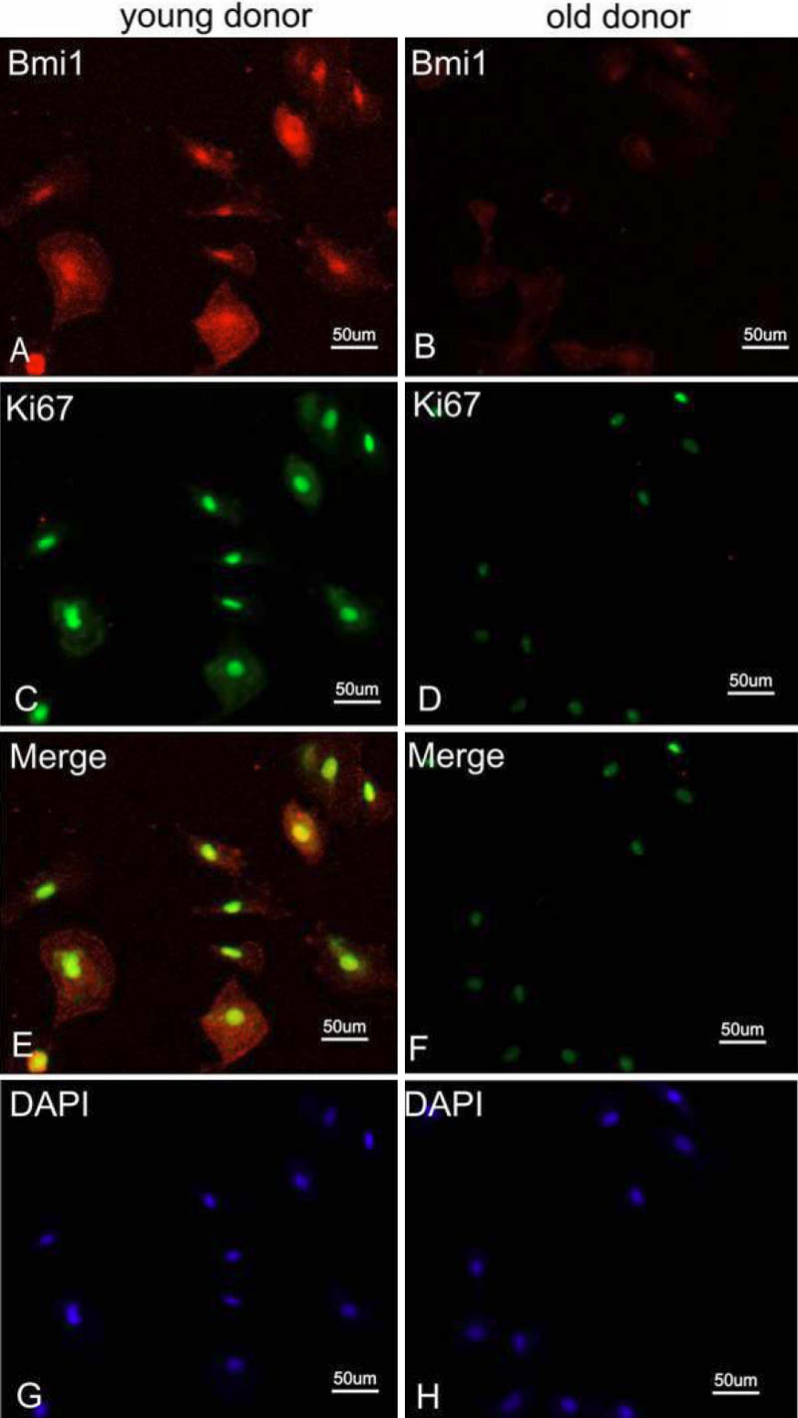
Results of double-staining of Bmi1 and Ki67. Immunofluorescence double-staining of Bmi1 (red) and Ki67 (green) in samples from a donor younger than 30 years of age (**A**, **C**, and **E**) and from a donor older than 50 years of age (**B**, **D**, and **F**). Panels **G** and **H** represent DAPI staining.

**Figure 10:**
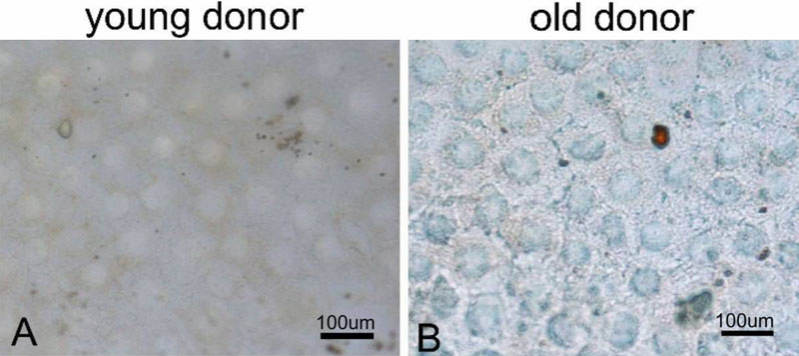
Results of SA-β-Gal activity staining. SA-β-Gal positive cells were not observed in HCECs from young donor (**A**), whereas SA-β-Gal positivity was observed in the HCECs from old donor (**B**).

For a better understanding of the relationship between cell cycle and two factors in HCEC senescence, double- staining of p16^INK4a^ and Bmi1 along with Ki67 in younger and older cases were performed. Figure 8 shows the results of co-staining of p16^INK4a^ and Ki67. The number of p16^INK4a^ - positive cells in HCECs from old donor were more than that in HCECs from young donor and few Ki67- positive cells were found in HCECs from old donor. Figure 9 shows the results of co-staining of Bmi1 and Ki67. The number and strength of Bmi1-positive cells in HCECs from young donor were higher than that in HCECs from old donor. Besides the expression of p16^INK4a^ and Bmi1 along with Ki67, we also detected the HCECs senescence using an independent marker such as SA-β-gal activity staining (Figure 10). We found few cells that were stained positive for SA-β-gal in HCECs from young donor (Figure 10A). However, many SA-β-gal positive cells were found in HCECs from old donor (Figure 10B).

## Discussion

Here, we have shown that the expression of *Bmi1* in HCECs and high expression of *p16^INK4a^* and low expression of *Bmi1* are associated with endothelial cellular senescence in human cornea. HCECs are arrested in early G_1_ phase in vivo [4,32,33]. The reason for this arrest is unknown, but the negative control of the corneal endothelial cell cycle has been found to increase in an age-dependent manner [34]. Compared with our previous report showing an age-related increase in *p16^INK4a^* expression in normal HCECs in vivo [18], in the present study, we increased the number of samples and expanded the age span investigated and these results were consistent. Findings from our laboratory have demonstrated that in the senescence-accelerated mouse (SAM), the increased expression of *p16^INK4a^* is also an age-dependent phenomenon [35]. In the present study, we have evaluated *Bmi1* and *p16^INK4a^* expression in human corneal endothelium biopsies from different age groups by immunohistochemistry and real-time PCR, which are relatively simple methods to measure changes in *Bmi1* and *p16^INK4a^* expression.

At the transcriptional level, the expression of *p16^INK4a^* is modulated by three principal regulators: v-ets erythroblastosis virus E26 oncogene homolog 1 (*EST1*), inhibitor of DNA binding 1 (*ID1*), and B lymphoma Mo-MLV insertion region (*Bmi1*) [36,37]. Previous studies have firmly established that *Bmi1* and *p16^INK4a^* are factors that control senescence in vitro [37,38]. In WI-38 human fetal lung fibroblasts, *Bmi1* is down-regulated when the cells undergo replicative senescence, but not when they are quiescent [39]. Lifespan extension by *Bmi1* is mediated in part by suppression of the *p16^INK4a^*-dependent senescence pathway [40]. In the absence of *Bmi1*, *p16^INK4a^* is upregulated and prevents binding of cyclin-dependent kinase 4/6 (Cdk4/6) to cyclin D, which inhibits the kinase activity of Cdk. Normal mouse embryonic fibroblasts (MEFs) from *Bmi1*^–/–^ mice exhibited a premature senescence phenotype that was correlated with increased expression of *p16^INK4a^* [37,39]. The close correlation between the upregulation of *Bmi1* and down-regulation of *p16^INK4a^* has been demonstrated in various tumors [19,41-43]. Current data show that *Bmi1* regulated the expression of *p16^INK4a^* by binding directly to the Bmi-1-responding element (BRE) within the *p16^INK4a^* promoter in a *Ring2* independent pathway [22]. However, the relevance of *p16^INK4a^* and *Bmi1* in the aging of HCECs in vivo remains unclear. In the present study, we have detected greater than twofold decreases in *Bmi1* expression with aging. To our knowledge, this is the first report indicating that *Bmi1* gene expression is significantly down-regulated in HCECs with increasing donor age. Because *Bmi1* is a negative regulator of *p16^INK4a^* gene expression, our data also represent the first evidence that *Bmi1* may control the *p16^INK4a^* gene expression in HCECs in vivo.

In summary, our data strongly support the possibility that *p16^INK4a^* can be used as a biomarker for HCECs senescence, as it is used for skin cells [16] and peripheral blood T-cells [15]. Additional studies are required to determine the *p16^INK4a^* expression pattern to elucidate the suitability of donor tissue for corneal transplantation. Our findings are not just for cornea transplantation but also for a better understanding of the cornea senescence and the process of aging in this sepcific human organ.
